# A novel somatic *BRCA2* point mutation in a metastatic pancreatic cancer patient: a case report

**DOI:** 10.1186/s12920-020-00850-6

**Published:** 2021-01-06

**Authors:** Deqiang Wang, Ruting Guan, Qing Tao, Sisi Liu, Man Yu, Xiaoqin Li

**Affiliations:** 1 Department of Medical Oncology, Affiliated Hospital of Jiangsu University, Zhenjiang, Jiangsu China; 2 Department of Research and Development, Nanjing Geneseeq Technology Inc., Nanjing, Jiangsu China; 3 Translational Medicine Research Institute, Geneseeq Technology Inc., Toronto, Canada

**Keywords:** *BRCA2*, Olaparib, Metastatic pancreatic cancer, PARP inhibitor, Somatic mutation

## Abstract

**Background:**

In addition to ovarian and breast cancers, loss-of-function mutations in *BRCA1* and *BRCA2* genes are also linked to an increased risk of pancreatic cancer, with ~ 4 to 7% of pancreatic cancer patients harboring germline *BRCA* mutations. Most *BRCA* alterations in pancreatic cancer are frame-shifting indels, stop-gain, and splice-site mutations, but single nucleotide substitutions are rare. Recent studies demonstrated a significant progression-free survival (PFS) benefit from maintenance olaparib, a poly (ADP-ribose) polymerase (PARP) inhibitor administered to patients with germline *BRCA* mutations and metastatic pancreatic cancer.

**Case presentation:**

Here, we report a metastatic pancreatic cancer case who harbored a novel somatic *BRCA2* c.6944T > C (p. I2315T) point mutation. After 6 weeks first-line chemotherapy, the patient was refractory to treatment and had a progressive disease. Due to the novel nonsynonymous *BRCA2* point mutation, we decided to change the strategy by administering olaparib. The patient benefited from olaparib therapy and achieved a PFS of ~ 6.5 months.

**Conclusions:**

We describe a patient carrying a novel somatic *BRCA2* p. I2315T point mutation, which is first reported in metastatic pancreatic cancer. This case report indicates that a gene mutation-based strategy should be considered in the clinic to provide more effective treatment.

## Background

Pancreatic cancer is the 7th leading cause of cancer-related deaths worldwide, with a 5-year survival rate of < 5% [1]. This devastating malignancy is generally diagnosed at metastatic stages in the clinic, indicative of its late detection and biological aggressiveness [1]. Tremendous efforts are ongoing with the aims of discovering early diagnostic markers and novel therapeutic avenues for pancreatic cancer; however, progress is remarkably hindered by the complicated heterogeneity within and between patient tumors.

*BRCA1* and *BRCA2* are essential players involved in homologous recombination repair of double-strand deoxyribonucleic acid (DNA) breaks [2]. *BRCA* inactivation due to somatic mutations or *BRCA1* promoter methylation have been observed in various cancer types, including a small subgroup of metastatic pancreatic cancer [3–5]. Most *BRCA* alterations in pancreatic cancer are frame-shifting indels, stop-gain, and splice-site mutations, whereas single nucleotide substitutions are rarely identified [2, 4, 5].

A large body of evidence showed that the poly (ADP-ribose) polymerase (PARP) inhibitors are synthetically lethal in *BRCA*-mutated tumors with DNA repair defects and displayed potent anti-tumor activity when combined with DNA-damaging agents. Thus, patients carrying germline mutations in *BRCA* or the various patterns of somatic *BRCA* mutations that could result in the inactivation of *BRCA* are sensitive to PARP inhibitors [6].

A recent phase 3 POLO (Pancreas Cancer Olaparib Ongoing) trial demonstrated that the administration of a PARP inhibitor, olaparib, as a maintenance therapy significantly prolonged the median progression-free survival (PFS) of patients with germline *BRCA* mutations and metastatic pancreatic cancer who had not progressed during platinum-based chemotherapy compared to the placebo arm (7.4 months vs. 3.8 months) [7]. More importantly, olaparib treatment did not compromise health-related quality of life in those patients [8]. However, PARP inhibitor resistance is common due to homologous recombination repair restoration (HRR), epigenetic modification, reversion mutations, restoration of ADP-ribosylation (PARylation), and pharmacological alteration [9].

In this report, we present the case of a metastatic pancreatic cancer patient who had progressive disease (PD) following chemotherapy with gemcitabine and nab-paclitaxel. Mutational profiling analysis using targeted next-generation sequencing (NGS) revealed that the patient carried a novel somatic *BRCA2* c.6944T > C (p. I2315T) point mutation. Olaparib was thereby administered in combination with a modified FOLFIRINOX regimen or as a monotherapy. The patient showed a significant response to this treatment strategy and exhibited stable disease and a PFS of ~ 6.5 months was observed.

## Case presentation

**Fig. 1:**
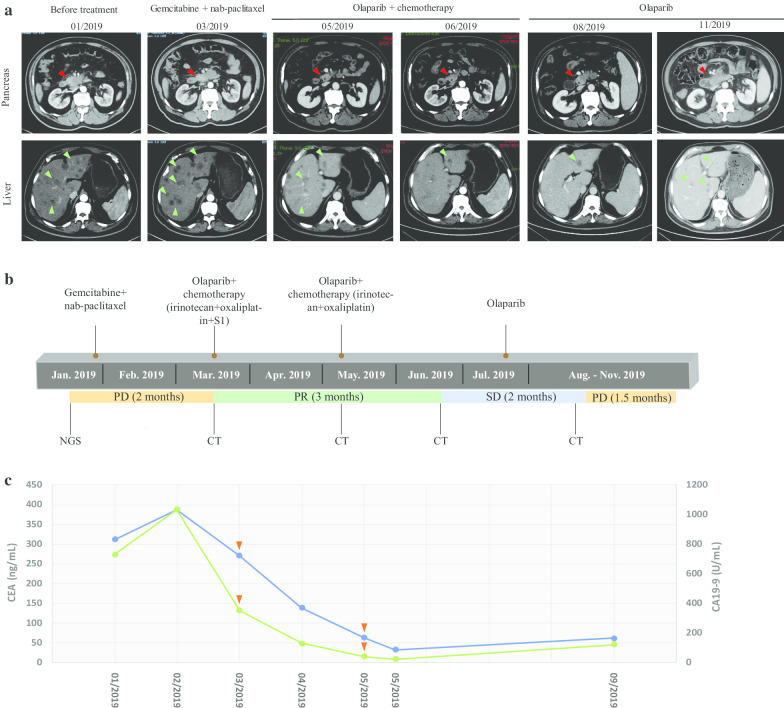
CT images and measurements of serum tumor biomarkers during the course of treatment. **a** Serial CT scans demonstrated a reduction in the size of the pancreatic (red arrows) and metastatic hepatic lesions (green arrows) following olaparib therapy. **b** A timeline indicating the application of different therapeutic strategies and the patient’s response. **c** Measurements of serum CEA and CA19-9 levels at different treatment times. Blue line: CEA; green line: CA19-9; orange arrows: time points of the CT scans. *PD* progressive disease, *PR* partial response, *SD* stable disease

A 57-year-old Chinese male with no obvious symptoms was admitted to the hospital due to the identification of a low-density shadow in the liver during his physical examination. In January 2019, a computed tomography (CT) scan revealed a 5.0 × 3.0 cm tumor in the uncinate process of the pancreas with hepatic metastases (Fig. 1a). The patient had a family history of cancer: his mother was diagnosed with stomach cancer at the age of 65 years and his father had lung cancer. According to the National Comprehensive Cancer Network (NCCN) Guidelines, the patient first received gemcitabine treatment at a dose of 1.4 g once per week, and nab-paclitaxel at a dose of 200 mg every 2 weeks as a first-line chemotherapy in January 2019 (Fig. 1b). Unfortunately, a CT re-evaluation at 6 weeks post-treatment showed progressive enlargement of both pancreatic and hepatic lesions (Fig. 1a), accompanied with a marked elevation of serum carcinoembryonic antigen (CEA, 270.35 ng/mL) and carbohydrate antigen 19-9 (CA19-9, 352.56 U/mL) levels (Fig. 1c); thus, suggesting that the patient was refractory to treatment and had a PD.

To determine a more effective and appropriate targeted therapy, we performed a targeted NGS analysis of 425 cancer-related genes on the patient’s plasma and tumor tissue biopsy samples. The cancer mutation panel test revealed multiple deleterious somatic mutations, such as the driver mutations, *KRAS* Q61R (c.182A > G), *TP53* R110del (c.329_331delGTC), and *APC* S1465RfsX9 (c.4393_4394dupAG), and copy number gain of several genes (Table 1). Interestingly, we also observed a novel nonsynonymous *BRCA2* c.6944T > C (p. I2315T) point mutation with a mutant allele frequency (MAF) of 28.0% in the plasma and 39.5% in the tumor biopsy specimens. The novel *BRCA2* point mutation was not detected in white blood cells, and thus, it was confirmed as a somatic mutation.

**Table 1:** Genetic alterations detected in the patient’s plasma and tumor biopsy specimens

Genes	Alternations	Nucleotide change	MAF (plasma)	MAF (tumor)
*BRCA2*	p. I2315T	c.6944T > C	28%	39.5%
*KRAS*	p. Q61R	c.182A > G	27.4%	43.2%
*TP53*	p. R110del	c.329_331delGTC	37.4%	81.6%
*APC*	p. S1465RfsX9	c.4393_4394dupAG	26.7%	71.7%
*CCNE1*	Gene amplification	NA	16.0-fold	27.4-fold
*CCNE1*	IGR (downstream UQCRFS1) ~ CCNE1 fusion	NA	0.1%	–
*PIK3CA*	Gene amplification	NA	–	1.9-fold
*PKHD1*	p. R909X truncation	c.2725C > T	4.9%	16.9%
*SOX2*	Gene amplification	NA	–	2.0-fold
*STMN1*	Gene amplification	NA	–	1.8-fold
*TERC*	Gene amplification	NA	–	1.9-fold
*TUBB3*	Gene amplification	NA	–	2.2-fold

–, not detectable; NA, not applicable; MAF, mutant allele frequency. Gene amplification was presented as the relative fold change to normal controls

Given the clinical efficacy of olaparib in treating *BRCA*-mutated advanced solid tumors, we decided to revise the therapeutic strategy by administering olaparib at a dose of 150 mg twice per day in combination with a modified FOLFIRINOX regimen (irinotecan at a dose of 100 mg on days 1, 8 and 15; oxaliplatin at a dose of 150 mg on days 1 and 15; S-1 at a dose of 40 mg twice per day from day 1 to day 14) for one cycle beginning in March 2019 (Fig. 1b). However, due to the occurrence of adverse events (e.g., diarrhea, general weakness, fatigue, and loss of appetite) and myelosuppression, including grade 2–3 leukopenia and thrombocytopenia, chemotherapy was not given on day 15 and the frequency of irinotecan was reduced to only be administered on days 1 and 15 in the subsequent treatment. Additionally, megestrol acetate and granulocyte-colony stimulating factor (G-CSF) were prescribed to increase appetite and promote the recovery of myelosuppression. In May 2019, a reduction in the size of both pancreatic and hepatic lesions was revealed by the CT scan, while the levels of the serum tumor biomarkers, CEA (63.38 ng/mL) and CA19-9 (40.53 U/mL) were considerably reduced (Fig. 1a, c). Olaparib and modified FOLFIRINOX were thus continued with irinotecan and oxaliplatin being administered only on day 1 (Fig. 1b). After 2 weeks of treatment, CEA and CA19-9 levels decreased to 32.69 ng/mL and 25.57 U/mL, respectively (Fig. 1c). A CT scan performed in June 2019 showed a significant size reduction in the primary and metastatic tumors, which indicated a partial response (PR, Fig. 1a). Since the patient experienced severe marrow suppression during treatment, only olaparib monotherapy was administered since July 2019 to reduce adverse effects (Fig. 1b). Stable disease (SD) was subsequently observed in August 2019 (Fig. 1a). In September 2019, aside from an increase in CEA and CA19-9 levels (Fig. 1c), laboratory blood tests demonstrated that the patient underwent acquired granulocytopenia, anemia, and thrombocytopenia. Consequently, G-CSF, thrombopoietin, and erythropoietin were administered for symptom management. After olaparib treatment for another 1.5 months, PD was indicated by a CT scan (Fig. 1a). The patient achieved a PFS for ~ 6.5 months following olaparib combination and monotherapy.

## Discussion and conclusions

Neoplastic cells lacking a functional homologous recombination repair system, such as those carrying *BRCA* mutations, are sensitive to PARP inhibition through accumulated DNA damage via multiple mechanisms [6]. As a new therapeutic concept, maintenance olaparib has shown promising results in the treatment of germline *BRCA*-mutated breast, ovarian, and metastatic pancreatic cancer [7, 10, 11]. To date, the majority of *BRCA2* mutations identified in pancreatic cancer were frame-shifting indels (e.g., c.6174delT, c.6158insT) and splice-site mutations. In contrast, single point mutations have been rarely reported [4, 5, 12]. Mesman [13] recently assessed the potential pathogenic impact of a large set of *BRCA2* missense variants using a mouse embryonic stem cell (mESC)-based functional assay, and found that *BRCA2* missense mutations, such as c.93G > T (p. W31C) and c.8351G > A (p. R2784Q), were able to increase susceptibility to PARP inhibitor treatment.

The patient in our report harbored an unreported *BRCA2* point mutation [c.6944T > C (p. I2315T)] located in exon 13 of *BRCA2* between the G-CSF. Different germline *BRCA2* mutations (e.g., p. I2315V, p. I2315L) at the same site have been documented with unknown functional significance [14, 15]. Although the underlying molecular mechanism(s) by which this novel *BRCA2* p. I2315T mutation impairs DNA repair and sensitizes tumor cells to PARP inhibition remains to be elucidated. We postulate that it may be related to a change in the polarity of amino acid residues since isoleucine (I), valine (V) and leucine (L) are all non-polar and hydrophobic, while threonine (T) is hydrophilic. Thus, we hypothesize that the p. I2315T mutation is likely to cause a structural abnormality in the *BRCA2* protein, which results in defective DNA double strand break (DSB) repair by homologous recombination (HR) and its sensitivity to olaparib. To test this hypothesis, we attempted to generate a three dimensional (3D) structural model of the *BRCA2* p. I2315T mutation, but failed due to the absence of an established crystal structure of the full-length human *BRCA2* due to its size and segmental nature [16].

In summary, we report a metastatic pancreatic cancer patient carrying a novel somatic *BRCA2* p. I2315T point mutation. Furthermore, advances in NGS technology have provided a solid basis for precise detection of well-known driver mutations, and rare or novel mutations. Thus, NGS may provide clinicians with invaluable information (e.g., *BRCA1/2* status of tumors) that can be leveraged for therapeutic decision making, and perform better evaluations of patients’ responses during the course of treatment.

## Data Availability

The datasets generated and/or analysed during the current study are not publicly available in order to protect participant confidentiality but are available from the corresponding author on reasonable request.

## Abbreviations

PARP: Poly(ADP-ribose) polymerase
PFS: Progression-free survival
PD: Progressive disease
DNA: Deoxyribonucleic acid
POLO: Pancreas cancer olaparib ongoing
CT: Computed tomography
NCCN: The National Comprehensive Cancer Network
CEA: Carcinoembryonic antigen
CA19-9: Carbohydrate antigen 19-9
NGS: Next-generation sequencing
MAF: Mutant allele frequency
FOLFIRINOX: FOL-folinic acid, F-fluorouracil, IRIN-irinotecan, OX-oxaliplatin
G-CSF: Granulocyte-colony stimulating factor
PR: Partial response
mESC: Mouse embryonic stem cell
DSBs: Double strand breaks
HR: Homologous recombination
3D: Three dimensional
